# Kinematics or Kinetics: Optimum Measurement of the Vertical Variations of the Center of Mass during Gait Initiation

**DOI:** 10.3390/s21237954

**Published:** 2021-11-29

**Authors:** Antoine Langeard, Charlotte Mathon, Mourad Ould-Slimane, Leslie Decker, Nicolas Bessot, Antoine Gauthier, Nathalie Chastan

**Affiliations:** 1 INSERM, COMETE, Université de Caen Normandie, 14000 Caen, France; leslie.decker@unicaen.fr (L.D.); nicolas.bessot@unicaen.fr (N.B.); antoine.gauthier@unicaen.fr (A.G.); Nathalie.Chastan@chu-rouen.fr (N.C.); 2 Department of Neurophysiology, Rouen University Hospital, 76000 Rouen, France; charlotte.mathon@gmail.com; 3 Spine Unit, Department of Orthopedic Surgery, Rouen University Hospital, 76000 Rouen, France; Mourad.Ould-Slimane@chu-rouen.fr

**Keywords:** kinematics, kinetics, braking index, dynamic balance, body center of mass

## Abstract

Background: During gait, the braking index represents postural control, and consequently, the risk of falls. Previous studies based their determination of the braking index during the first step on kinetic methods using force platforms, which are highly variable. This study aimed to investigate whether determining the braking index with a kinematic method, through 3D motion capture, provides more precise results. Methods: Fifty participants (20 to 40 years) performed ten trials in natural and fast gait conditions. Their braking index was estimated from their first step simultaneously using a force platform and VICON motion capture system. The reliability of each braking index acquisition method was assessed by intraclass correlation coefficients, standard error measurements, and the minimal detectable change. Results: Both kinetic and kinematic methods allowed good to excellent reliability and similar minimum detectable changes (10%). Conclusion: Estimating the braking index through a kinetic or a kinematic method was highly reliable.

## 1. Introduction

Falls, gait, and balance disorders represent a genuine public health concern due to their high prevalence in the aging population and patients suffering from neurological diseases [1,2]. They cause significant morbidity and mortality, functional deterioration, and earlier institutionalization [3,4]. Half of falling incidents occur during walking [5], and while many studies have already shown an interest in spatiotemporal gait parameters and posturography [6,7], changes in these parameters could result from adaptive strategies aimed at securing gait. They may, therefore, not be an optimal reflection of postural control abilities [8]. Only a few studies have evaluated dynamic postural control during gait by evaluating center of mass (CoM) vertical displacement (orthogonal axis to the plane corresponding to the floor), which is believed to more appropriately reflect postural control [9]. More specifically, during gait initiation (transition from standing posture to walking), CoM displacement has been used to differentiate healthy from disabled subjects [9].

In our previous studies, we specifically showed that the vertical displacement of the CoM reflects postural control during gait initiation [10,11,12,13,14]. Indeed, braking the falling CoM before foot contact can be described as an active mode of CoM control [10]. In healthy people, the velocity of the displacement of the CoM in the vertical axis is significantly reduced before foot contact. The central nervous system prepares for foot contact by decreasing the vertical velocity of the CoM to achieve a soft landing [10]. It has been reported that the most challenging point for stability is the instant before the contact of the swing leg [9]. For the movement to continue, the CoM velocity must be reduced to avoid falling. This reduction in velocity is called the CoM braking. This translates to a V-shaped representation of the vertical velocity of the CoM (VzCoM) during the swing phase—i.e., between foot off (FO) and foot contact (FC). The braking index (BI) is quantified by expressing the VzCoM at FC (V2) as a function of its absolute maximum value (V1), as follows [14]:

(V1 − V2)/V1,(1)

A BI closer to 1 indicates an important deceleration of the vertical velocity of the CoM before FC, while a BI closer to 0 indicates an absence of a deceleration of the vertical velocity of the CoM before FC.

Consequently, in all healthy adults, the increase in the BI value is concomitant with the increased activity of the soleus muscle of the stance leg [12]. Determining whether enhancing soleus muscle function through resistance training may benefit the BI remains to be tested and could represent a possible intervention in patients suffering from pathological declines in BI performances. Among patients with progressive supranuclear palsy [14], which is a parkinsonian syndrome characterized by the presence of severe postural instability, and among patients with Parkinson’s disease with altered balance [11], no braking occurred, which means that the vertical velocity was not reduced before FC.

Moreover, natural or fast gait conditions led to similar braking capacities [11]. These different studies showed that the BI accurately represents balance control during gait [15]. These previous studies all used kinetic methods through force platform evaluations. Indeed, force platforms use strain gage technology, which provides force and moment components along the axes of an orthogonal x, y, z-coordinate system, allowing CoM estimations to be made from these ground reaction forces. This kinetic measurement method showed a high intra-individual variability of the BI values [16].

Whether the BI measure with three-dimensional kinematic methods, in which CoM location estimation is based on a commonly used 3D model (Plug-in-Gait, Vicon) relying on a marker-based optical motion capture [17], would lead to a more reliable measurement is not known. The kinematic method allows CoM displacement to be directly measured, but the kinetic method allows foot-related events to be direct measured, both pieces of information being needed to estimate the BI. 

The present study’s main aim was to determine which of the two acquisition methods, kinematics or kinetics, produced the highest test–retest reliability. The secondary aim of this study was to determine if the two methods provided comparable measures of gait velocity and step length. We hypothesized that the kinematic method might produce a higher reliability in comparison to the kinetic method.

## 2. Materials and Methods

### 2.1. Participants

Fifty participants, from 20 to 40 years old (29 women; mean age—26.9 years (SD—5.5); mean height—172.6 cm (SD—9.9); mean weight—70.6 kg (SD—14.8)), took part in this study conducted at Rouen University Hospital, France. Participants were recruited through posters displayed in the hospital. Inclusion criteria were: healthy participants aged between 20 and 40 years old, and use of an effective contraceptive method for women of childbearing age. Exclusion criteria were: neurologic, rheumatologic, vestibular or orthopedic disease, any spinal or skeleton deformation, severe visual disorders, incapacity to walk without any assistance, and pregnant or nursing women. All participants gave written informed consent, and the study was approved by the local ethics committee (NCT02231827).

### 2.2. Walking Test

Thirty-nine reflective markers were positioned on anatomic points of participants in underwear (participants were only wearing underwear), following the Plug-in-Gait full-body model (Vicon Motion Systems, Oxford, UK) [17]. Standing upright and barefoot on a force platform, participants were instructed to walk 5 steps following the experimenter signal. Two experimental conditions were tested: natural gait (self-selected speed) and fast gait (maximal speed). Ten trials were performed for the natural gait condition, and ten trials were performed for the fast gait condition. The acquisition was performed simultaneously for both methods (force platform and optoelectronic system) for the first step, between standing posture and the first foot contact. The following four steps were not recorded by both methods and, therefore, were not analyzed

The force platform (Advanced Mechanical Technology Inc., LG6-4-1, USA, 0.9 m × 1.8 m) allowed the recording of the first step [11]. It provided continuous signals proportional to the ground reaction forces (Rx, Ry, and Rz in Newtons) and moments (Mx, My, and Mz in Newton meters) on the mediolateral (X), anteroposterior (Y), and vertical (Z) axes of the force plate. The force plate analog signals were sampled at a frequency of 1000 Hz. According to Newton’s law, these signals allowed us to obtain the displacement of the Center of Pressure (CoP) and the accelerations of the CoM. By dividing Mx by Rz and Ry by the subject’s body mass and (Rz − BodyWeight) by the body mass, we obtained the anteroposterior displacement of the CoP and the anteroposterior and vertical CoM accelerations [11], respectively:

Mx/Rz = anteroposterior displacement of the CoP,(2)

Ry/body mass = anteroposterior CoM acceleration,(3)

(Rz − BodyWeight)/body mass = vertical CoM acceleration,(4)

The optoelectronic system was a VICON T10/6 system (Oxford Metrics Ltd., Oxford, UK) using six MXT10 cameras (1 million pixels) [18]. This method allowed us to provide the displacement in time of all body segments. Combined with the anthropometric data, this information allowed the automatic calculation of the displacement of the body CoM through the Vicon Plug-in-Gait model (Nexus 1.8.5, Oxford Metrics UK). The signals were digitized at 100 Hz [17].

All signals were recorded and calculated using a GIGANETLAB unit core, motion capture software VICON Nexus 1.8.5, and automation server software PECS 1.1.60305.

### 2.3. Biomechanical Analysis

In order to compare BI measurement reliability, both the kinematic and the kinetic methods were used to determine the BI during the first step. The BI was then calculated in the same way for both methods and corresponded to:

BI = (V1 − V2)/V1,(5)

with V1 being the minimal value of VzCoM (Equation (6)) between FO and FC (the negative peak of the CoM vertical velocity) and V2 the value of VzCoM at FC.

On the force platform, VzCoM was calculated using the vertical acceleration defined by:

VzCoM = (Fz/m) − (Fzt0/m),(6)

where m = mass, Fz = weight, Fzt0 = weight at t0 when the participant is standing still on the platform, and FC was determined by the sudden anterior displacement of the CoP. With the optoelectronic system, FC was determined by visualizing the position of the heel marker in relation to the ground (FC was at the lowest position of the heel marker, right before the heel marker changed trajectory). For both methods, step length (L) corresponded to the distance between 2 FC and Gait speed (Vm), represented by the maximum CoM instantaneous progression velocity reached at the end of the step.

A few trials were not usable and were excluded from the statistical analyses (i.e., if the participant stumbled or ran or if there was a drift from the baseline of the VzCoM with the force platform). When participants were not moving at t0, Fz was equal to Fzt0, so the velocity was constant, and no drift occurred. On the other hand, if participants were moving, Fzt0 was superior or inferior to Fz. Therefore, velocity was not constant, and a baseline drift was occurring.

Little’s Missing Completely at Random test showed that the missing data were missing completely at random for kinematic (Chi-squared = 107.390, DF = 94, Sig. = 0.163) and kinetic data (Chi-squared = 649.001, DF = 681, Sig. = 0.806). Missing data were therefore assumed to not matter for the analysis.

### 2.4. Statistical Analysis

Reasonable precision for reliability estimates requires approximately 50 study participants and at least three trials [19]. Therefore, the sample size, the number of trials, and the evaluation method correspond to what is advised in this field [19]. The reliability of the BI measure was assessed by calculating the intercorrelation coefficients (ICC), standard error of measurement (SEM), and minimum detectable changes (MDC) for each acquisition method in both normal and fast conditions. The ICC and 95% confidence intervals were calculated using SPSS 26.0 (Tulsa, OK, USA) based on absolute agreement and 2-way mixed-effect (ICC < 0.90: excellent reliability; 0.75 < ICC < 0.9: good reliability; 0.5 < ICC < 0.75: moderate reliability; 0.5 > ICC: poor reliability) [20]. The SEM corresponded to the square root of the within-subjects error variance, and the MDC = 1.96 × SEM × $\sqrt{2}$ [21]. Changes below the MDC can be attributed to measurement errors.

The effect of the acquisition method, kinematic or kinetic, on the gait parameters was evaluated through a two-way (methods of acquisition × trial condition) repeated-measure analysis of variance (ANOVA). Tukey’s post hoc analysis was processed when the ANOVA detected an interaction between factors. Conditions of validity were tested with the Shapiro–Wilk and the Mauchly tests.

## 3. Results

The mean and CV of the parameters of interest (BI, Vm, and L), recorded using the kinematic and the kinetic methods, are presented in Table 1.

Only 49 of the overall 50 participants were included in the analysis because, for one participant, the force platform data recorded were unusable. In the natural gait condition, the ICC of the BI measures were 0.902 (95%IC = 0.860, 0.940) and 0.905 (95%IC = 0.856, 0.938), for the kinematic and the kinetic methods, respectively. In the fast gait condition, the ICC were 0.919 (95%IC = 0.881, 0.949) and 0.870 (95%IC = 0.808, 0.918), for the kinematic and the kinetic methods, respectively. The reliability of both methods can therefore be considered to be between good to excellent [20]. The SEM values for the BI measures in normal gait conditions were 0.037 and 0.041 for the kinetic and kinematic methods, respectively, resulting in MDC values of 0.103 and 0.114. In fast gait conditions, the SEM values were 0.038 and 0.0409 for the kinetic and kinematic methods, respectively, resulting in MDC values of 0.106 and 0.113. The differences of the MDC of BI between acquisition methods were therefore estimated to be around 1%. The results of the ANOVA are presented in Table 2.

The repeated-measure ANOVA detected differences between the acquisition methods (kinetic vs. kinematic) in BI (*p* < 0.001), Vm (*p* = 0.006), and L (*p* < 0.001) measures. Differences between the trial conditions (normal vs. fast gait) were also detected for Vm (*p* < 0.001) and L (*p* < 0.001) measures. For the natural gait conditions, compared to the kinematic method, the kinetic method led to a 26% lower BI, a 24% higher Vm, and a 8% lower L. For the fast gait conditions, the kinetic method led to a 21% lower BI, a 31% higher Vm, and a 10% lower L. 

## 4. Discussion

The main hypothesis of the present study was that the kinematic method would produce a more reliable measurement of the BI during gait initiation than the kinetic method. However, this hypothesis was not validated by the results. Indeed, both methods led to highly reliable measures of BI and similar MDC. This confirms the robustness of the previous studies in terms of the BI measured through kinetic acquisition [10,11,12,13,14,15,22,23,24] and highlights that future studies could also use kinematic analyses to produce equivalent reliable measures. Accordingly, a previous study comparing the vertical excursion of the CoM between the two acquisition methods (kinematics and kinetics), based on a small sample (*n* = 10), did not detect any difference between these methods [25].

In addition, a difference in the mean values of the gait parameters recorded through the two measurement methods was detected, which could represent a measurement error that is consistent between trials in one or both of the acquisition methods. In this study, one possible factor involved in this possible measurement error of the kinetic measure was the imprecise manual determination of the FC obtained from the anteroposterior or the mediolateral center of the foot pressure displacement curve. Indeed, while a kinematic analysis can spatially detect the exact position of the foot in relation to the ground and therefore calculate the L and the BI, the FC is manually estimated on the force platform through the analysis of the curves representing the forces during gait, and this could depend on how the foot hits the ground. This led to a variation in V2 and the BI. The imprecision related to kinetic measurements has already been brought to light [26], and the use of kinematics has already been suggested to avoid errors linked to extrapolations of the calculus of the CoM position [17]. Regardless, this evaluation of FC is validated in the literature [9,10,11,12,14,15,22,26].

Lowering the intra-individual variability allows not only a more precise measurement of the BI, but could also allow a reduction in the number of trials necessary in order to achieve a meaningful measure [27]. This could counteract the time-consuming procedure associated with kinematic measurements (preparation of the participants, anthropometrical measurements, calibration, virtual labeling of body markers, etc.). Nevertheless, intra-individual variability is an intrinsic parameter of gait [28]. The BI, in particular, seems to be naturally highly variable among individuals. The reasons and implications of this high intra-individual variability should be further studied in order to provide a better understanding of postural control. Of interest, the BI has recently been shown to be affected by cognitive decline and could become a cognitive-related gait marker to estimate fall risk without the addition of cognitive tasks [29]. BI has also been shown to be related to chronic ankle instability [30]. Future studies should confirm the possibility for the BI to become an early marker for cognitive or motor decline.

This study is the first to determine that the kinematic measure of the BI is more precise than the kinetic method. Further studies should determine if combining these different methods could lead to a higher reliability. While there was a need to validate this hypothesis in healthy adults, further studies should also determine if our conclusion is valid for participants who are prone to falling or present gait impairments.

## 5. Conclusions

Evaluating the BI through a kinematic or kinetic analysis allows highly reliable measures. This finding confirms the validity of the previous findings based on kinetic analysis and validates the use of kinematic analysis in the estimation of the BI. This could bring new perspectives to the analysis and understanding of gait and balance disorders in relation to cognitive or motor impairments.

## Figures and Tables

**Table 1:** Gait parameters during gait initiation measured with the kinematic and kinetic methods (mean (sd)).

	Natural Gait Condition		Fast Gait Condition	
	Kinematics	Kinetics	Kinematics	Kinetics
Braking Index (%)	69.9 (18.5)	52.0 (21.0)	68.9 (19.6)	54.3 (18.9)
Gait velocity (m·s^−1^)	0.99 (0.12)	1.05 (0.13)	1.52 (0.16)	1.51 (0.14)
Step length (cm)	61.3 (6.1)	56.1 (6.0)	72.4 (8.9)	65.4 (8.7)

**Table 2:** Results of the two-way (trial condition × acquisition method) repeated-measure ANOVA on braking index, gait velocity, and step length during gait initiation.

		Mean Square	*p*
Braking Index	Acquisition method	1.312	<0.001
	Trial condition (natural or fast)	0.002	0.771
	Interaction: method × condition	0.014	0.207
Gait velocity	Acquisition method	0.030	0.006
	Trial condition (natural or fast)	12.353	<0.001
	Interaction method × condition	0.052	<0.001
Step Length	Acquisition method	177,890.02	<0.001
	Trial condition (natural or fast)	507,554.87	<0.001
	Interaction method × condition	4083.12	<0.001

## Data Availability

The authors confirm that the data supporting the findings of this study will be made available upon request.
